# Histopathology of Diabetic Nephropathy: Beyond Glomerular Basement Membrane Thickening

**DOI:** 10.7759/cureus.93497

**Published:** 2025-09-29

**Authors:** Hussein Qasim, Shaima' Dibian, Anas Hayajneh, Karis Khattab, Matteo Luigi Giuseppe Leoni, Giustino Varrassi

**Affiliations:** 1 Department of Pathology and Laboratory Medicine, Jordan University of Science and Technology, Irbid, JOR; 2 Faculty of Medicine, Jordan University of Science and Technology, Irbid, JOR; 3 Department of Medical and Surgical Sciences and Translational Medicine, Sapienza University, Rome, ITA; 4 Department of Pain Medicine, Fondazione Paolo Procacci, Rome, ITA

**Keywords:** arteriolar hyalinosis, diabetic nephropathy, glomerular basement membrane thickening, histopathology, mesangial expansion, nodular glomerulosclerosis, podocyte injury, tubulointerstitial fibrosis

## Abstract

Diabetic nephropathy (DN) is a leading cause of chronic kidney disease and end-stage renal disease worldwide, characterized by progressive injury across all renal compartments. Traditionally considered a glomerular disorder marked by basement membrane thickening, mesangial expansion, and nodular sclerosis, DN is now recognized as a multifaceted process involving tubulointerstitial, vascular, and podocyte pathology. This narrative review integrates current histopathological insights, highlighting key glomerular lesions, tubular basement membrane thickening, interstitial fibrosis, arteriolar hyalinosis, and podocyte injury, as well as atypical and advanced patterns. The role of kidney biopsy in diagnosis, the application of the Renal Pathology Society classification, and differentiation from mimicking conditions such as amyloidosis and monoclonal deposition disease are discussed. Emerging biomarkers and advanced imaging modalities offer promise for earlier detection and risk stratification. Understanding the full histological spectrum of DN is critical for accurate diagnosis, prognostication, and the development of targeted therapies. Future directions include molecular classification, non-invasive diagnostics, and precision medicine approaches to improve patient outcomes.

## Introduction and background

Diabetic nephropathy (DN) is one of the leading causes of chronic kidney disease (CKD) and end-stage renal disease (ESRD) worldwide, representing a significant clinical and economic burden on healthcare systems [1]. It affects approximately 20-40% of individuals with diabetes mellitus and is associated with increased cardiovascular morbidity and mortality [2]. As the global prevalence of diabetes continues to rise, particularly in low- and middle-income countries, the incidence of DN is expected to increase in parallel [3].

DN, a microvascular complication of chronic hyperglycemia, involves progressive kidney changes, from early glomerular hyperfiltration to proteinuria and declining filtration rate [4]. Although early stages may be clinically silent, histopathologic changes appear well before nephropathy and are influenced by genetic, metabolic, and hemodynamic factors [5,6].

Traditionally, DN has been viewed predominantly as a glomerular disease, with classical histological findings including thickening of the glomerular basement membrane (GBM), mesangial expansion (increased deposition of matrix material within the supporting mesangial areas of the glomerulus), and eventually the formation of nodular glomerulosclerosis, known as Kimmelstiel-Wilson nodules [6]. These changes reflect the combined effects of hyperglycemia-induced metabolic stress, hemodynamic forces, and growth factor-mediated matrix accumulation [7].

However, emerging evidence underscores that DN is a multifaceted disease process involving all compartments of the kidney [8]. Beyond glomerular lesions, significant alterations occur in the tubulointerstitial space and the renal vasculature [9]. Tubular basement membrane (TBM) thickening, interstitial inflammation, and progressive fibrosis (scarring of the tissue between renal tubules) are now recognized as key contributors to renal functional decline [10]. Similarly, arteriolar hyalinosis and arteriosclerosis reduce perfusion and promote ischemic injury [11]. Another critical component is podocyte injury and loss, a process now known to play a pivotal role in the development of proteinuria and glomerulosclerosis [11].

Expanding the histopathological focus beyond glomerular lesions is crucial, as tubulointerstitial and vascular changes often correlate more closely with renal function decline and may guide earlier, more targeted therapeutic interventions [11]. This narrative review provides a comprehensive overview of the histopathology of DN, moving beyond the classic glomerular paradigm to explore the full range of structural changes.

## Review

Methodology

This narrative review was conducted using a structured and transparent approach designed to enhance rigor and reproducibility. The review followed the Scale for the Assessment of Narrative Review Articles (SANRA) guidelines to ensure methodological quality [12]. A comprehensive literature search was performed across PubMed, Scopus, Embase, Web of Science (WoS), and Google Scholar. Search terms were constructed using Medical Subject Headings (MeSH) and Boolean operators to maximize both precision and breadth. The primary search string was (“diabetic nephropathy” OR “diabetic kidney disease”) AND (“histopathology” OR “renal biopsy” OR “glomerular lesions” OR “tubulointerstitial fibrosis” OR “vascular lesions” OR “podocyte injury” OR “classification” OR “differential diagnosis”). Eligible literature included peer-reviewed articles focusing on the histopathological features, classification systems, differential diagnosis, emerging biomarkers, and clinical correlations of DN. Accepted study types encompassed original research articles, systematic reviews, meta-analyses, narrative reviews, case series, and authoritative textbook chapters. Only publications available in full-text English were considered. Studies were excluded if they lacked relevance to histopathological or diagnostic aspects of DN, did not provide interpretive context, or were not peer-reviewed. Reference lists of relevant articles were manually screened to identify additional studies not captured by the database searches. The final selection of literature was synthesized thematically to present an integrated overview of the morphological spectrum, diagnostic frameworks, and future directions in DN research. To reduce bias, we excluded non-peer-reviewed studies, conference abstracts, and articles lacking sufficient methodological detail. Only full-text English publications were considered.

Pathogenesis of DN

The pathogenesis of DN is multifactorial, primarily driven by chronic hyperglycemia and altered renal hemodynamics [13]. Prolonged hyperglycemia activates a range of metabolic and inflammatory pathways, including the formation of advanced glycation end-products (AGEs), oxidative stress, and the upregulation of cytokines and fibrogenic growth factors, such as transforming growth factor beta (TGF-β) [14]. These processes promote abnormal cell signaling in glomerular and tubular cells, leading to excessive extracellular matrix deposition and thickening of both glomerular and TBMs [15]. Hemodynamic factors contribute further, with early diabetes causing glomerular hyperfiltration and increased intraglomerular pressure, resulting in endothelial injury and glomerular hypertrophy [16]. Activation of the renin-angiotensin-aldosterone system (RAAS) and mechanical stress further exacerbate capillary wall remodeling and sclerosis [17]. Inflammation also plays a pivotal role, with hyperglycemia promoting macrophage infiltration and pro-inflammatory cytokine release that amplifies tissue injury [18]. Podocytes, critical for maintaining the filtration barrier, are especially vulnerable to hyperglycemia, and AGEs lead to podocyte dysfunction, detachment, and apoptosis, contributing to progressive glomerulosclerosis [19]. The earliest and most characteristic histopathological changes in DN are GBM thickening and diffuse mesangial expansion [5]. GBM thickening can precede clinical signs such as proteinuria and is identifiable via electron microscopy (EM) as a diffuse accumulation of matrix proteins such as type IV collagen [20]. Mesangial expansion, driven by hyperglycemia and TGF-β, typically becomes evident after several years of diabetes and is associated with increased albuminuria and declining kidney function [21]. These two often coexist and form the cornerstone of classical diabetic glomerulopathy [21].

Tubulointerstitial changes

While glomerular lesions are central to DN, tubulointerstitial changes are now recognized as critical contributors to disease progression [22]. In fact, the extent of tubulointerstitial injury often correlates more strongly with renal functional decline than glomerular damage alone [23]. One key feature is TBM thickening, which mirrors GBM changes [24]. In both proximal and distal tubules, basement membranes become diffusely thickened due to glycation and excessive matrix deposition [25]. This is detectable on EM and special stains in LM and may impair tubular reabsorptive function early in the disease [25]. Tubular atrophy is another hallmark of DN. Prolonged hyperglycemia and increased tubular reabsorption of filtered proteins lead to epithelial injury, apoptosis, and dedifferentiation [26]. Atrophic tubules appear shrunken with flattened epithelium and thickened TBMs, contributing to nephron dropout and reduced functional capacity [27]. Interstitial fibrosis develops in response to tubular injury and chronic inflammation [28]. Characterized by the accumulation of type I and III collagen in the interstitial space, it is driven by sustained expression of profibrotic factors such as TGF-β and by tissue hypoxia from downstream capillary rarefaction [29]. Fibrosis is typically patchy in early DN but becomes more widespread in advanced stages, and its severity is a strong predictor of glomerular filtration rate (GFR) decline [30]. In parallel, inflammatory cell infiltrates, mainly macrophages and lymphocytes, accumulate in areas of fibrosis or surrounding injured tubules [31]. These cells release cytokines and chemokines that sustain tissue injury and promote further fibrosis [32]. Notably, inflammation may be present even in areas without obvious scarring, suggesting active and ongoing injury [33].

Vascular lesions

Diabetes mellitus is well known for causing microangiopathy, and the renal vasculature exhibits characteristic changes in this context. Both small arterioles and larger arteries supplying the kidney are affected. Arteriolar hyalinosis is a hallmark lesion of DN, involving both afferent and efferent arterioles. Histologically, these vessels show thickened, glassy walls due to the deposition of eosinophilic hyaline material, composed of plasma proteins and glycosylated matrix, that accumulates within the vessel wall. The presence of efferent arteriolar involvement, which is uncommon in benign hypertension, is considered virtually pathognomonic for DN. This process leads to luminal narrowing and downstream ischemia affecting both glomeruli and the tubulointerstitial compartment. Arteriosclerosis, involving the interlobular and arcuate arteries, is also common in DN. It is characterized by intimal thickening and fibroelastic hyperplasia, often to the point where the intima exceeds the media in thickness. Diabetes accelerates this process, leading to vessel stiffening and impaired parenchymal perfusion. In advanced DN, both arteriolar hyalinosis and arterial sclerosis are typically present. Other vascular lesions, including glomerular microaneurysms and exudative changes such as fibrin caps and capsular drops, may also be observed. These lesions reflect capillary wall damage and plasma protein leakage, resulting in hyaline deposits within glomerular lumens or beneath Bowman’s capsule. Functionally, these vascular abnormalities cause chronic ischemic injury, which accelerates tubular atrophy and interstitial fibrosis. This, in turn, contributes to glomerulosclerosis and further compromises renal perfusion. Clinically, these changes often mirror similar microvascular complications in other organs, such as the retina, underscoring the systemic nature of diabetic microangiopathy.

Podocyte pathology

Podocytes, specialized epithelial cells lining the outer aspect of glomerular capillaries, are essential for maintaining the integrity of the glomerular filtration barrier [34]. In DN, podocytes are among the earliest and most critical targets of injury, undergoing structural, molecular, and functional alterations [35]. Foot process effacement is one of the earliest detectable changes [36]. Under diabetic conditions, cytoskeletal disruptions lead to broadening and flattening of the interdigitating foot processes, disrupting slit diaphragm integrity [37]. On electron microscopy, this appears as a smooth, continuous layer where discrete foot processes should be [38]. This effacement correlates with increased glomerular permeability and proteinuria, serving as a non-specific but important indicator of podocyte injury [39]. Podocyte loss (podocytopenia) follows sustained injury [40]. Hyperglycemia and AGEs induce oxidative stress and downregulate vital proteins like nephrin and podocin, leading to podocyte apoptosis or detachment from the GBM [41]. Because podocytes have limited regenerative capacity, their depletion is essentially permanent [42]. The loss of these cells leaves the GBM exposed, promoting glomerular scarring and segmental sclerosis [12]. The extent of podocyte loss is strongly associated with proteinuria severity and disease progression, particularly in type 2 diabetes [43]. Phenotypic changes also occur before overt loss [44]. Podocytes may de-differentiate, expressing markers of injury (e.g., desmin) and losing slit diaphragm proteins [45]. Under hyperglycemic stimulation, they may also overproduce extracellular matrix components, including collagen IV and laminin, thereby contributing to GBM thickening and mesangial expansion [46].

Advanced and atypical patterns

As DN progresses, both glomerular and extraglomerular lesions can become more severe and may develop into advanced or atypical histological patterns [47]. One of the hallmark advanced glomerular lesions is nodular glomerulosclerosis, classically known as Kimmelstiel-Wilson nodules [48]. These are large, acellular, eosinophilic mesangial nodules composed of extracellular matrix, often located centrally within the glomerulus, displacing surrounding capillary loops [49]. They stain strongly with periodic acid-Schiff (PAS) and silver methenamine stains due to their rich collagen content [50]. Although not present in every case, their identification, typically in 20-30% of advanced DN biopsies, is considered pathognomonic when correlated with clinical evidence of diabetes [51]. Nodular glomerulosclerosis is associated with long-standing disease, marked proteinuria, significant renal impairment, and often coexisting diabetic retinopathy [52]. These nodules tend to enlarge and may merge, ultimately leading to global glomerular sclerosis and glomerular obsolescence [53]. Other advanced lesions include widespread global glomerulosclerosis, particularly in end-stage diabetic kidney disease, where most glomeruli are completely scarred [54]. Exudative lesions, such as fibrin caps, hyaline deposits lining the interior of glomerular capillaries, and capsular drops, hyaline accumulations beneath Bowman’s capsule, may also be observed [55]. These changes reflect capillary wall injury and plasma protein leakage, and while not exclusive to DN, they are considered characteristic histologic features in advanced disease [56]. In contrast, not all diabetic patients display the classic pattern of glomerular-predominant injury [57]. Some exhibit atypical histological presentations in which tubulointerstitial or vascular lesions are disproportionately severe relative to glomerular changes [58]. This is particularly common in individuals with type 2 diabetes, where renal biopsies may reveal marked arteriolar hyalinosis and extensive interstitial fibrosis, yet only minimal mesangial expansion [49]. These cases are sometimes referred to as “atypical diabetic nephropathy” and may resemble hypertensive nephrosclerosis [59]. The older Fioretto classification recognized such variants, categorizing them as “C3”, cases where arteriolar or tubulointerstitial lesions predominated over glomerular involvement [60]. Another atypical scenario involves the coexistence of superimposed renal injuries, such as acute tubular necrosis or renal papillary necrosis, which can occur alongside chronic diabetic changes [61]. Additionally, focal segmental glomerulosclerosis (FSGS)-like lesions may develop due to glomerular hyperfiltration and podocyte loss, especially in patients with obesity or long-standing diabetes [62]. These segmental scars typically affect the periphery of the glomerular tuft and may mimic primary FSGS histologically [63].

Diagnostic tools

Diagnosing DN typically begins with clinical suspicion in a patient with diabetes, especially when there is persistent albuminuria, a slowly progressive decline in renal function, and concurrent diabetic retinopathy [64]. However, definitive diagnosis and characterization of histological changes require a kidney biopsy, particularly in cases with atypical features [65]. Light microscopy (LM) remains the cornerstone of histopathologic evaluation [66]. Several stains are routinely used to highlight specific renal structures [67]. Hematoxylin and eosin (H&E) provides a general overview and can reveal eosinophilic nodules or thickened arteriolar walls [68]. PAS stain enhances visualization of the mesangial matrix and thickened GBM, often highlighting diabetic nodules in a magenta hue [69]. Jones' methenamine silver stain is especially useful for delineating basement membranes and accentuating the lace-like pattern of Kimmelstiel-Wilson nodules, as well as confirming GBM thickening [49]. Trichrome stain, which colors collagen blue, is valuable for assessing interstitial fibrosis and global glomerular sclerosis [70].When these features, diffuse or nodular glomerulosclerosis, arteriolar hyalinosis, and interstitial fibrosis, appear together in a diabetic patient, they strongly support the diagnosis of DN [71]. Immunofluorescence (IF) is employed to detect immune complex deposition or light chain involvement [72]. In classical DN, IF is typically negative or shows only nonspecific linear IgG staining along the GBM and TBMs, likely due to binding of IgG to AGEs [71]. This linear staining is of low intensity and should not be mistaken for that seen in anti-GBM disease [73]. Occasionally, mild IgM or C3 staining may be observed in sclerotic areas, again reflecting nonspecific trapping rather than immune complex deposition [74]. Importantly, true immune complexes are absent in DN, and their presence should raise suspicion for superimposed glomerulonephritis [75]. When nodular lesions are present, IF should also be used to rule out amyloidosis by evaluating for light chain restriction, and Congo red staining should be performed to exclude amyloid deposition [76]. Moreover, EM plays a crucial role, particularly in early or ambiguous cases [77]. EM allows precise measurement of GBM thickness and can detect characteristic irregular or lucent areas reflecting matrix glycation [78]. It also enables direct visualization of podocyte foot process effacement, a hallmark of early podocyte injury in DN [79]. Critically, EM can confirm the absence of electron-dense immune deposits, which distinguishes DN from other immune complex-mediated glomerulopathies [80]. Additionally, EM reveals changes in the TBMs, such as thickening and intracellular glycogen accumulation, further supporting the diagnosis [81]. Although laboratory and clinical assessments are not histologic tools, they are essential for diagnostic context [82]. Persistent albuminuria, particularly when gradually increasing, is a clinical hallmark of DN, often developing after years of diabetes [83]. Hematuria is typically absent; its presence, especially if accompanied by red blood cell casts, may suggest another glomerular pathology [83]. The presence of diabetic retinopathy, particularly in type 1 diabetes, strongly supports a diagnosis of DN [84]. Conversely, if kidney disease occurs in a diabetic patient without retinopathy, especially early in the course of diabetes, a biopsy should be considered to rule out a non-diabetic kidney disease (NDKD) [85]. In clinical practice, not every diabetic patient with renal impairment undergoes biopsy, as DN is often diagnosed presumptively [65]. However, a renal biopsy is indicated when there are atypical features such as sudden onset of nephrotic syndrome, rapid decline in the GFR, active urinary sediment with numerous cells or casts, or unusually early onset of renal disease relative to diabetes duration [86]. In such cases, biopsy can help differentiate pure DN from coexisting or alternative glomerulopathies [87]. When performed and interpreted using LM, IF, EM, and clinical correlation, renal biopsy provides a definitive histopathologic diagnosis and guides appropriate management [88].

Classification systems

To stratify the histologic severity of DN, pathologists have developed classification systems [89]. The most widely used is the Renal Pathology Society (RPS) classification published by Tervaert et al. in 2010 [90]. This system focuses on glomerular changes and provides a standardized way to categorize DN lesions from mild to advanced. In addition, it includes separate grading of tubulointerstitial and vascular lesions. Table 1 summarizes the RPS pathologic classification of DN.

**Table 1:** Pathologic Classification of Diabetic Nephropathy (Renal Pathology Society (RPS) 2010) The table was reproduced from Tervaert et al. [88] with permission. EM: electron microscopy; GBM: glomerular basement membrane; LM: light microscopy

Class	Glomerular Lesion (Description and Criteria)
I	Mild or nonspecific LM changes; GBM thickening only (confirmed by EM). GBM width > ~430 nm in men or > ~395 nm in women (greater than normal range).
IIa	Moderate mesangial expansion in >25% of the tuft area (mild diabetic glomerulopathy). Mesangial area increased but not exceeding capillary lumen area.
IIb	Severe mesangial expansion in >25% of tuft (marked diffuse glomerulosclerosis). Mesangial areas are expanded to the point that mesangial matrix exceeds capillary luminal area.
III	Nodular diabetic glomerulosclerosis. At least one Kimmelstiel-Wilson nodule in one or more glomeruli. (Diffuse mesangial expansion typically also present.)
IV	Advanced diabetic glomerulopathy. >50% of glomeruli are globally sclerosed (obsolescent). Often accompanied by severe tubulointerstitial and vascular lesions.

While RPS 2010 standardizes the histologic reporting of DN, it also acknowledges that glomerular lesions alone may not fully capture disease severity [90]. To address this, the RPS also includes a semiquantitative scoring system for tubulointerstitial and vascular lesions, which are reported alongside the glomerular class [90]. Tubulointerstitial damage is graded from 0 to 3 based on the percentage of cortical involvement with interstitial fibrosis and tubular atrophy (IFTA): 0 for none, 1 for <25%, 2 for 25-50%, and 3 for >50%. Interstitial inflammation is graded separately, 0 if absent, 1 if confined to fibrotic areas, and 2 if present in non-fibrotic regions as well [91]. Vascular lesions are also scored: arteriolar hyalinosis is graded 0 if absent, 1 if one arteriole is involved, and 2 if more than one arteriole is affected. Arteriosclerosis is scored as 0 for no intimal thickening, 1 if the intima is less than the thickness of the media, and 2 if it exceeds the media [91]. For example, a pathology report might describe a case as “Class III DN with IFTA score 2, arteriolar hyalinosis 2, arteriosclerosis 1,” providing a more comprehensive picture of renal damage. Prior to the RPS system, earlier frameworks attempted to capture the morphologic heterogeneity of DN [92]. Notably, the Fioretto classification, developed in the 1990s, divided DN into three categories: Class I for normal or near-normal histology, Class II for typical diabetic glomerulopathy (with either diffuse or nodular changes), and Class III for “atypical” forms where tubulointerstitial or vascular injury predominated despite minimal glomerular abnormalities [93]. This approach was particularly relevant for type 2 diabetes, where atypical patterns are more frequently observed [93]. While the Fioretto system emphasized the broader range of diabetic renal pathology, the current RPS classification focuses primarily on glomerular lesions and has faced some criticism for insufficiently capturing extraglomerular damage in certain patients. Looking ahead, efforts are underway to refine classification further by incorporating molecular profiling and more nuanced histological data [94]. One such initiative is the TRIDENT (Transformative Research in Diabetic Nephropathy) study, which aims to develop an integrative system that combines histopathology with transcriptomic and proteomic signatures to improve outcome prediction [95]. Despite these emerging approaches, the Tervaert/RPS classification remains the gold standard for pathological assessment of DN [96]. It provides a uniform and widely accepted language for pathologists and clinicians to characterize the stage and severity of diabetic kidney disease beyond what clinical parameters alone can reveal [96].

Differential diagnosis and mimickers

The histopathological appearance of DN, particularly in its advanced nodular form, can be mimicked by several other renal diseases [5]. Accurate differentiation is essential, as these conditions differ significantly in treatment and prognosis [5]. Renal amyloidosis is a key consideration [97]. In particular, AL amyloidosis can produce glomerular nodules that superficially resemble Kimmelstiel-Wilson lesions seen in DN [98]. However, amyloid nodules differ in staining characteristics: they are Congo Red-positive with apple-green birefringence under polarized light and tend to be PAS-negative or weak, in contrast to the PAS- and silver-positive matrix nodules of DN [99]. Amyloid deposits also typically involve not only glomeruli but also vessels and the interstitium [100]. IF may show monoclonal light chain (often λ) deposits, and EM confirms the diagnosis by revealing characteristic non-branching, randomly arranged fibrils approximately 10 nm in diameter [101]. Because of this overlap, Congo Red staining is mandatory whenever nodular glomerulosclerosis is identified on biopsy [102]. Light chain deposition disease (LCDD) and other monoclonal immunoglobulin deposition diseases also mimic diabetic nodular lesions [103]. In LCDD, monoclonal light chains (typically kappa) deposit along the GBMs and TBMs, often staining positively with PAS and silver stains, closely resembling DN [103]. However, IF reveals linear staining with monoclonal light chains, and EM shows fine granular, “powdery” electron-dense deposits rather than the matrix expansion of DN [103]. Patients with LCDD usually do not have diabetes and often have underlying plasma cell dyscrasias [104]. Related diseases, such as fibrillary glomerulonephritis, can also produce nodular glomerular patterns, but these are characterized by immune complex deposition on IF and fibrils measuring around 20 nm on EM, clearly distinct from DN [105]. Another important mimic is idiopathic nodular glomerulosclerosis (ING), a rare entity with pathology strikingly similar to diabetic nodular glomerulosclerosis but occurring in non-diabetic patients [106]. ING has been most commonly described in heavy smokers with long-standing hypertension and is thought to result from smoking-induced AGE accumulation and vascular injury that mimics diabetic damage [107]. These patients often have normal blood glucose levels but may exhibit impaired glucose tolerance [108]. The diagnosis is one of exclusion, made after ruling out diabetes, amyloidosis, and monoclonal deposition diseases [109]. Several other glomerular diseases may occasionally present with mesangial or nodular lesions [109]. Membranoproliferative glomerulonephritis (MPGN), although primarily a proliferative disorder, can form mesangial nodules accompanied by mesangial hypercellularity, double-contoured capillary walls, and immune deposits, all features that help distinguish it from DN [110]. Cryoglobulinemic glomerulonephritis may feature pseudo-nodules formed by large immune deposits, identifiable through IF and EM by their substructure [111]. Fibronectin glomerulopathy, an extremely rare hereditary condition, can also produce PAS-positive but silver-negative nodules, diagnosable by special immunostains for fibronectin [112]. It is equally important to recognize that patients with diabetes may develop concurrent non-diabetic renal diseases (NDRD) [112]. In biopsy studies of type 2 diabetics with atypical clinical presentations, a substantial proportion have been found to harbor NDRD either alone or superimposed on DN [113]. Common examples include IgA nephropathy, membranous nephropathy, FSGS (especially obesity-related), and acute interstitial nephritis [114]. Clinical signs that raise suspicion for NDRD include active urinary sediment (e.g., dysmorphic red blood cells or red blood cell casts), sudden onset of nephrotic syndrome, rapid deterioration of renal function, or absence of diabetic retinopathy [115]. For instance, a patient with heavy proteinuria but no retinopathy may have membranous nephropathy rather than DN [116]. Sometimes DN and NDRD coexist, requiring recognition of both to guide management, especially since some NDRDs may warrant immunosuppressive therapy, a treatment not used for isolated DN [117]. In practical terms, the differential diagnosis of DN relies on careful integration of clinical context with histological findings [118]. When nodular glomerulosclerosis is observed, it is standard practice to perform Congo Red staining, comprehensive IF (for immunoglobulins and complement), and EM to distinguish between DN and other nodular glomerular diseases [119]. These tools ensure diagnostic accuracy and help clinicians choose appropriate treatment pathways (Table 2).

**Table 2:** Differential Diagnosis of Nodular Glomerulosclerosis DN: diabetic nephropathy; EM: electron microscopy; IF: immunofluorescence; GBM: glomerular basement membrane; MPGN: membranoproliferative glomerulonephritis; PAS: periodic acid-Schiff

Condition	Key Distinguishing Features (vs. DN)
Diabetic Nephropathy (DN) [119]	Nodules with PAS(+), silver(+) matrix; IF: ± linear IgG (non-specific); Congo Red negative; afferent & efferent arteriolar hyalinosis present; clinical diabetes present (often with retinopathy).
AL Amyloidosis [120]	Nodules are Congo Red positive (apple-green birefringence); PAS and silver often negative; IF: monoclonal light chain (λ>κ) deposits; EM: 10 nm fibrils. Glomeruli often enlarged. Can have other organ involvement (heart, etc.).
Light Chain Deposition (LCDD) [121]	Nodules PAS(+) & silver(+); IF: monoclonal light chain (κ) linear deposits in GBM/TBM; EM: granular electron-dense deposits (“powdery”) in nodules and along GBM. Often no diabetes; patient has plasma cell disorder.
Idiopathic Nodular GS [106]	Identical glomerular lesions to DN (PAS+, silver+ nodules, arteriolar hyalinosis) but the patient is not diabetic. Often a history of heavy smoking & hypertension. No amyloid or monoclonal deposits on workup.
Others (e.g., MPGN, Fibrillary) [122,123]	MPGN: nodules with hypercellularity and immune deposits (IF positive, EM dense deposits). Fibrillary GN: may have nodular appearance but IF shows polyclonal IgG and EM shows larger fibrils (~20 nm). Typically distinguishable by detailed immunopathology.

Emerging biomarkers

Traditional markers, such as albuminuria and serum creatinine, detect DN only after substantial kidney damage has occurred [124]. To enable earlier detection and better risk stratification, several novel biomarkers are being investigated, reflecting injury across different renal compartments [125]. Tubular injury markers, such as neutrophil gelatinase-associated lipocalin (NGAL) and kidney injury molecule-1 (KIM-1), rise early in DN, even before albuminuria, signaling proximal and distal tubular stress [125]. Other tubular proteins, such as NAG and L-FABP, may reflect oxidative stress and early tubulointerstitial dysfunction [126]. Inflammatory and fibrotic markers are also promising [126]. Circulating soluble TNF receptors (sTNFR1 and sTNFR2) are strong predictors of disease progression, while monocyte chemoattractant protein-1 (MCP-1) in urine correlates with interstitial inflammation [127]. TGF-β1, a fibrosis mediator, is under study but not yet clinically reliable [128]. To assess glomerular and podocyte injury, urinary levels of podocyte proteins such as nephrin and podocin and circulating suPAR levels are being explored [129]. These may indicate podocyte stress or depletion before major glomerular lesions become visible [129]. Because DN involves extracellular matrix expansion, markers of matrix remodeling such as urinary type IV collagen, matrix metalloproteinases (MMPs), and periostin are also under evaluation as indicators of fibrogenesis [130]. Newer approaches using “omics” technologies, such as urinary microRNAs and metabolomics, are identifying distinct molecular signatures associated with early DN or rapid progression [131]. Finally, advanced imaging techniques such as DCE-MRI and PET with targeted tracers offer the potential to visualize renal fibrosis and inflammation noninvasively, though they remain experimental [132].

Clinical and therapeutic implications

Understanding the histopathology of DN is crucial for guiding diagnosis, prognosis, and treatment [133]. The severity of lesions, ranging from early GBM thickening to nodular sclerosis and global glomerulosclerosis, correlates with disease progression and renal outcomes [134]. Patients with early changes may enjoy prolonged kidney function with optimal glycemic and blood pressure control, while those with advanced lesions face a higher risk of end-stage renal disease [135]. Biopsy becomes particularly valuable when atypical features suggest a superimposed pathology, allowing tailored management and avoiding unnecessary treatments [136]. Emerging therapies targeting specific pathways, such as SGLT2 inhibitors and finerenone, are supported by histologic evidence and help mitigate both renal and cardiovascular risks [137]. Beyond diagnostics, histologic findings can shape therapeutic decisions. Proteinuria, for example, often indicates podocyte injury, warranting RAAS blockade [138]. Histology also plays a powerful role in patient education; seeing structural damage can enhance adherence and inform expectations [139]. Ultimately, histopathology bridges pathology with practice, shaping a more precise and personalized approach to diabetic kidney care [140].

Future directions

The future of DN research is poised to transform both diagnosis and management through a more nuanced understanding of its pathogenesis [13]. One major direction is the refinement of classification systems to better capture disease heterogeneity [141]. Current histological frameworks, while helpful, may soon be complemented by integrated approaches that incorporate molecular, transcriptomic, and proteomic data [142]. Efforts such as the TRIDENT study aim to develop precision medicine models, identifying distinct subtypes of DN based on underlying biological mechanisms, such as inflammatory, metabolic, or fibrotic profiles, which could help tailor therapies to individual patients more effectively [143]. Non-invasive diagnostics also represent a critical frontier [144]. As biopsies remain invasive and infrequently performed, future strategies may rely on advanced imaging modalities and liquid biopsies [145]. Techniques such as MRI elastography or PET imaging could provide real-time visualization of renal fibrosis or microvascular changes, while urinary exosomes and circulating biomarkers may offer insights into tissue-level processes without the need for histological sampling [146]. While MRI elastography shows promise for non-invasive assessment of renal fibrosis, its use is limited by motion artifacts, technical demands, cost, and restricted availability [146]. These innovations could enable earlier detection and closer monitoring of disease progression [147]. Linked to these diagnostics is the rise of biomarker-driven early intervention [148]. As promising biomarkers such as KIM-1, NGAL, or soluble TNF receptors undergo validation, they may allow identification of high-risk individuals before irreversible nephron loss [149]. Although promising, the cost-effectiveness of implementing these biomarkers in routine practice is not yet established, and large-scale validation studies are required before widespread clinical adoption [149]. Rather than applying biomarker panels universally, their use may be more appropriate in high-risk subgroups, patients with long-standing diabetes, poor glycemic control, hypertension, albuminuria or reduced eGFR, and coexisting diabetic retinopathy, where the predictive value and clinical impact are likely greatest [150]. Therapeutically, several novel avenues are under active investigation. Antifibrotic agents targeting TGF-β or other fibrogenic pathways may address the interstitial injury that drives long-term progression [151]. Compounds that inhibit or reverse AGE accumulation are being optimized to reduce structural damage to basement membranes and vessels [152]. Podocyte-protective strategies, including agents that stabilize the cytoskeleton or enhance autophagy, offer potential to preserve the integrity of the glomerular filtration barrier [153]. In parallel, regenerative medicine approaches, such as stem cell therapies or podocyte replacement, are being explored, albeit still in experimental stages [154]. Emerging insights into the gut-kidney axis and microbiome also open potential new interventions aimed at modulating systemic inflammation and metabolism [155]. Artificial intelligence is beginning to reshape renal pathology [156]. Digital pathology platforms, equipped with machine learning algorithms, can quantify structural lesions such as GBM thickening or interstitial fibrosis more precisely and reproducibly than manual methods [157]. These tools not only enhance diagnostic accuracy but may also facilitate predictive modeling by integrating histologic features with clinical parameters [158]. Finally, addressing DN on a global scale demands attention to health disparities and public health infrastructure [159]. Many patients, especially in low-resource settings, experience delayed diagnosis and limited access to proven therapies such as SGLT2 inhibitors [160]. Expanding screening efforts, improving access to care, and promoting early lifestyle and pharmacologic interventions remain essential components of reducing the burden of DN worldwide [6].

## Conclusions

DN is a complex, multifaceted disease that extends well beyond the classical paradigm of GBM thickening. Modern histopathological insights reveal that virtually all compartments of the kidney, including the glomeruli, tubulointerstitium, vasculature, and podocytes, undergo progressive injury in the diabetic milieu. Recognizing the full spectrum of these lesions is essential for accurate diagnosis, timely differentiation from other renal pathologies, and guiding effective clinical management. Kidney biopsy, when indicated, provides invaluable prognostic and therapeutic information, particularly in atypical or rapidly progressive cases. As our understanding deepens through advances in molecular biology, imaging, and biomarker discovery, the future of DN care is shifting toward more precise, individualized approaches. Continued integration of histologic findings with emerging diagnostic and therapeutic strategies holds promise not only for delaying disease progression but also for transforming how diabetic kidney disease is understood and managed across diverse patient populations.
